# Nitrate Starvation Induces Lateral Root Organogenesis in *Triticum aestivum* via Auxin Signaling

**DOI:** 10.3390/ijms25179566

**Published:** 2024-09-03

**Authors:** Chengming Tang, Yunxiu Zhang, Xiao Liu, Bin Zhang, Jisheng Si, Haiyong Xia, Shoujin Fan, Lingan Kong

**Affiliations:** 1 College of Life Science, Shandong Normal University, Jinan 250014, China; 2022020865@stu.sdnu.edu.cn (C.T.); 2021020839@stu.sdnu.edu.cn (X.L.); 2 Crop Research Institute, Shandong Academy of Agricultural Sciences, Jinan 250100, China; 13116012750@163.com (Y.Z.); vb5337@126.com (B.Z.); sijisheng114@163.com (J.S.); haiyongxia@cau.edu.cn (H.X.)

**Keywords:** wheat, nitrate, lateral root, indole-3-acetic acid, cell wall

## Abstract

The lateral root (LR) is an essential component of the plant root system, performing important functions for nutrient and water uptake in plants and playing a pivotal role in cereal crop productivity. Nitrate (NO_3_^−^) is an essential nutrient for plants. In this study, wheat plants were grown in 1/2 strength Hoagland’s solution containing 5 mM NO_3_^−^ (check; CK), 0.1 mM NO_3_^−^ (low NO_3_^−^; LN), or 0.1 mM NO_3_^−^ plus 60 mg/L 2,3,5-triiodobenzoic acid (TIBA) (LNT). The results showed that LN increased the LR number significantly at 48 h after treatment compared with CK, while not increasing the root biomass, and LNT significantly decreased the LR number and root biomass. The transcriptomic analysis showed that LN induced the expression of genes related to root IAA synthesis and transport and cell wall remodeling, and it was suppressed in the LNT conditions. A physiological assay revealed that the LN conditions increased the activity of IAA biosynthesis-related enzymes, the concentrations of tryptophan and IAA, and the activity of cell wall remodeling enzymes in the roots, whereas the content of polysaccharides in the LRP cell wall was significantly decreased compared with the control. Fourier-transform infrared spectroscopy and atomic microscopy revealed that the content of cell wall polysaccharides decreased and the cell wall elasticity of LR primordia (LRP) increased under the LN conditions. The effects of LN on IAA synthesis and polar transport, cell wall remodeling, and LR development were abolished when TIBA was applied. Our findings indicate that NO_3_^−^ starvation may improve auxin homeostasis and the biological properties of the LRP cell wall and thus promote LR initiation, while TIBA addition dampens the effects of LN on auxin signaling, gene expression, physiological processes, and the root architecture.

## 1. Introduction

Nitrogen (N) is an essential nutrient for plant growth and development and is involved in various metabolic processes [1,2]. Nitrate (NO_3_^−^) and ammonium (NH_4_^+^) are the main mineral forms of N that plants utilize from the external environment [3]. In particular, besides NO_3_^−^ being a macroelement, NO_3_^−^ signaling can regulate numerous biological processes, root organogenesis, organ growth, and development in plants [4,5,6,7]. However, an increasing number of studies have demonstrated that the irrational use of N fertilizers, especially one-off basal fertilization at planting, often causes soil acidification [8,9,10], environmental pollution, and plant growth inhibition and increases production costs, thus leading to low N use efficiency (NUE) [11]. 

Biotic and abiotic stress frequently occur during plant growth and development. To adapt to severely fluctuating environments, plants often evolve compensation mechanisms. In particular, the root system is the primary organ that senses and responds to environmental changes, besides anchoring the plant and absorbing water and nutrients [12]. Typically, the root systems of higher plants are mainly composed of primary and lateral roots (LRs) and show high plasticity when subjected to various ambient factors [13,14]. For instance, when exposed to a localized source of NO_3_^−^, the root system exhibits high plasticity by proliferating LRs in higher plants [15,16]. Deficiencies in potassium and phosphorus (Pi) negatively impact root elongation [17,18,19]. However, it has also been observed that Pi starvation enhances LR development and root hair emergence and elongation [20,21]. In Arabidopsis, primary root and LR development were inhibited by manganese due to the disruption of auxin biosynthesis and transport [22]. Boron deficiency decreased root growth by disturbing IAA biosynthesis [23,24]. Therefore, studying the responses of the root system to interactions between nutrients and auxin is of great significance in understanding plant development.

LRs are major architectural determinants of the root system and display higher plasticity in response to external water and nutrient availability [12,25]. Plant hormones play crucial roles in regulating LR formation and growth. In *Arabidopsis thaliana*, gibberellins were shown to positively control root apical meristem growth and LR formation [26,27]. Ethylene, an important gaseous phytohormone, exerted an inhibitory effect on LR formation, showing an antagonistic function with auxin in this process [25]. Cytokinins disrupted the LR patterning process by targeting LR founder cells [28]. Auxin, the first hormone discovered in plants, is involved in all stages of plant growth and development [29], including the regulation of root meristem activity and vascular tissue differentiation [30,31] and LR initiation and elongation [32,33]. In addition, enhanced auxin transport through various types of transporters promotes LR growth in plants [14,33,34,35]. Fine-tuning between auxin production and transport as well as inactivation is a prerequisite for LR positioning and development [17,36]. During LR formation, auxin signaling is specifically involved in the regulation of the coordinated asymmetric cell division of xylem-pole pericycle cells, periclinal cell division, and LR primordium (LRP) building, as well as setting the pace to facilitate LRP emergence [37,38]. 

Wheat (*Triticum aestivum* L.) is an important food crop around the world and occupies an extremely important position in food security. However, the mechanisms underlying roots’ responses to N starvation are not fully understood in wheat. The objectives of this study were to investigate the underlying mechanisms through which low levels of NO_3_^−^ regulate LR development and to propose regulatory networks for the interactions between low NO_3_^−^ and auxin in regulating LR development. This study thus provides a better understanding of root morphogenesis and aids in exploring reasonable N applications for wheat production.

## 2. Results

### 2.1. Plant Growth

**Figure 1:**
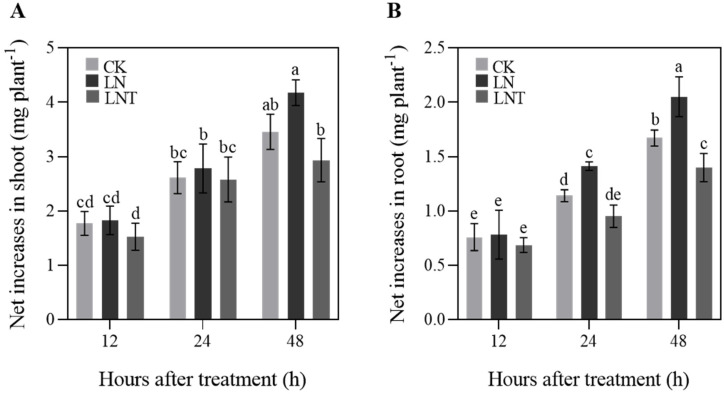
Net increases in dry weight of shoots (**A**) and roots (**B**) of wheat seedlings grown in solution containing 5 mM NO_3_^−^ (CK), 0.1 mM NO_3_^−^ (LN), and 0.1 mM NO_3_^−^ + 60 mg/L TIBA (LNT) at 12, 24, and 48 h after treatment. Values represent the mean ± SD of at least three independent experiments. Different letters above columns indicate significant differences at *p* ≤ 0.05.

No significant difference in the net increase in the shoot dry weight of wheat seedlings was observed in 5 mM NO_3_^−^ (CK), 0.1 mM NO_3_^−^ (LN), or 0.1 mM NO_3_^−^ plus 60 mg/L 2,3,5-triiodobenzoic acid (TIBA) (LNT) at 12 and 24 h (Figure 1A). At 24 and 48 h after treatment, the net increase in the dry weight of LNT and CK roots was significantly lower than that of LN, and no significant difference in the net increase in the root dry weight was observed between CK, LN, and LNT at 12 h (Figure 1B). At 48 h after treatment, the net increase in the dry weight of LNT shoots was significantly lower than that of LN, and no significant difference in the net increase in the shoot dry weight was observed between the CK and LN treatments (Figure 1A).

### 2.2. Root System Architecture

**Figure 2:**
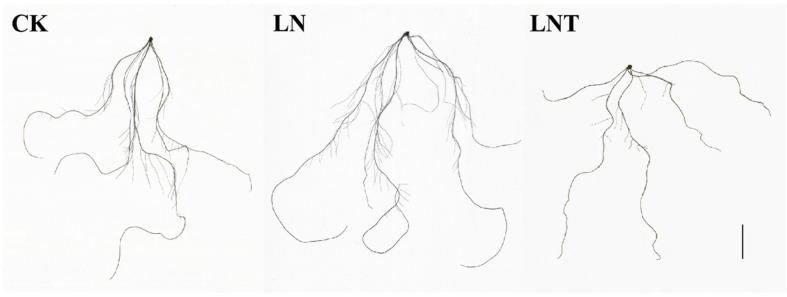
Representations of the root morphology of wheat seedlings at 48 h after treatment with 5 mM NO_3_^−^ (CK), 0.1 mM NO_3_^−^ (LN), and 0.1 mM NO_3_^−^ + 60 mg/L TIBA (LNT). Bar = 2 cm.

The wheat seedlings grown in LN conditions showed substantial changes in root morphogenesis compared with the CK plants (Figure 1A). Namely, the total root tips, total root length, and total surface area (*p* < 0.05) of the LN seedlings increased by 44.81% (*p* < 0.05), 20.34% (*p* < 0.05), and 14.16% (*p* < 0.05), respectively. The addition of TIBA decreased these parameters by 58.78% (*p* < 0.05), 48.12% (*p* < 0.05), and 39.49% (*p* < 0.05), respectively, compared to the LN conditions (Table 1). These data indicate that LN significantly increases LR development and thus increases the total root length and root surface area; however, the positive effect of low NO_3_^−^ on root morphology is mitigated after the addition of TIBA (Figure 2). 

### 2.3. Differentially Expressed Genes (DEGs) under Different N Treatments

A total of 7519.96 million genes were obtained, with average GC content of approximately 55.00%, Q20 of 98.40%, and Q30 of 95.40% and an average base sequencing error rate of 0.01, yielding 35,741,272 (95.04%) clean sequences. Combining the GC content and Q30, we concluded that the data were highly accurate and reliable for further experiments and subsequent analysis.

**Figure 3:**
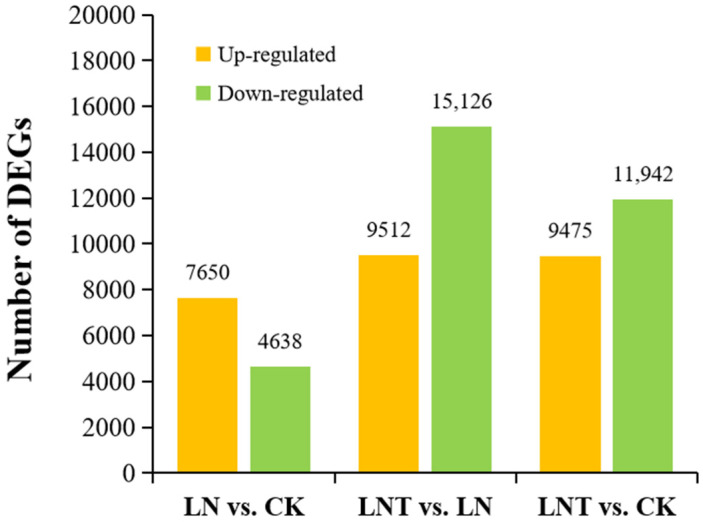
Paired comparisons of DEGs between different N treatments. Wheat seedlings grown in solution containing 5 mM NO_3_^−^ (CK), 0.1 mM NO_3_^−^ (LN), and 0.1 mM NO_3_^−^ + 60 mg/L TIBA (LNT) at 48 h after treatment.

A differential analysis was performed using the DESeq2 package and employing a screening criterion of |log_2_(FoldChange)| ≥ 1 and *p* < 0.05. In the comparison between LN and CK, 12,288 DEGs were identified, with 7650 DEGs upregulated and 4638 DEGs downregulated. In LNT vs. LN, 24,638 DEGs were identified, with 9512 DEGs upregulated and 15,126 DEGs downregulated (Figure 3). In contrast, of the 21,417 DEGs in the LNT vs. CK comparison, only 11,942 genes were downregulated and 9475 were upregulated. These data indicate that the roots strongly respond to LN conditions, and the responses are strongly inhibited upon the addition of TIBA. 

### 2.4. GO and KEGG Analysis of Differentially Expressed Genes

**Figure 4:**
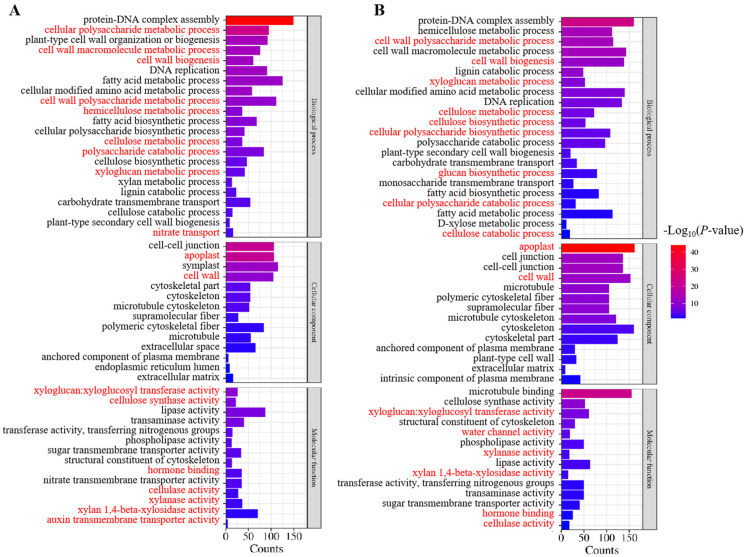
GO analysis of DEGs for the roots of wheat seedlings treated with 5 mM NO_3_^−^ (CK), 0.1 mM NO_3_^−^ (LN), and 0.1 mM NO_3_^−^ + 60 mg/L TIBA (LNT) for 48 h. (**A**) LN vs. CK; (**B**) LNT vs. LN. The *X*-axis indicates the number of differential genes annotated for the GO count, and the *Y*-axis indicates the enriched GO terms. The color gradient from red to blue represents the significance level of the −Log_10_(*p*-value). The key functions of DEGs that were involved in auxin signaling, cell wall remodeling, nitrogen mentalism, and water transport have been highlighted using the red font.

Based on the functional annotations, the DEGs between the CK, LN, and LNT libraries were classified into three Gene Ontology (GO) categories: biological process (BP), cellular component (CC), and molecular function (MF). The GO cellular component analysis showed that, in the comparison between the two groups of LN and CK, the processes associated with the BP group mainly included the cell wall polysaccharide metabolic process, the fatty acid metabolic process, protein–DNA complex assembly, DNA replication, plant-type cell wall organization or biogenesis, the cellular polysaccharide metabolic process, and plant-type cell wall organization. The DEGs of the CC group were mainly associated with microtubules, the extracellular space, polymeric cytoskeletal fibers, cell walls, apoplasts, cell–cell junctions, symplasts, and the microtubule cytoskeleton. Finally, the DEGs of the MF group were divided into nitrate transmembrane transporter activity, hormone binding, auxin transmembrane transporter activity, cellulase activity, xylanase activity, and xylan 1,4-beta-xylosidase activity (Figure 4A). In LNT vs. LN, the main enriched DEGs in the BP group were associated with protein–DNA complex assembly, the hemicellulose metabolic process, the cell wall polysaccharide metabolic process, the cell wall macromolecule metabolic process, and cell wall biogenesis. The DEGs in the CC group were mainly classified as apoplasts, cell–cell junctions, cell walls, and the cytoskeleton. The main categories in the MF group included structural constituents of the cytoskeleton, xyloglucan–xyloglucosyl transferase activity, lipase activity, transferase activity, the transfer of nitrogenous groups, cellulase activity, and microtubule binding (Figure 4B). These results suggest that low NO_3_^−^ conditions affect a variety of physiological processes in wheat roots.

**Figure 5:**
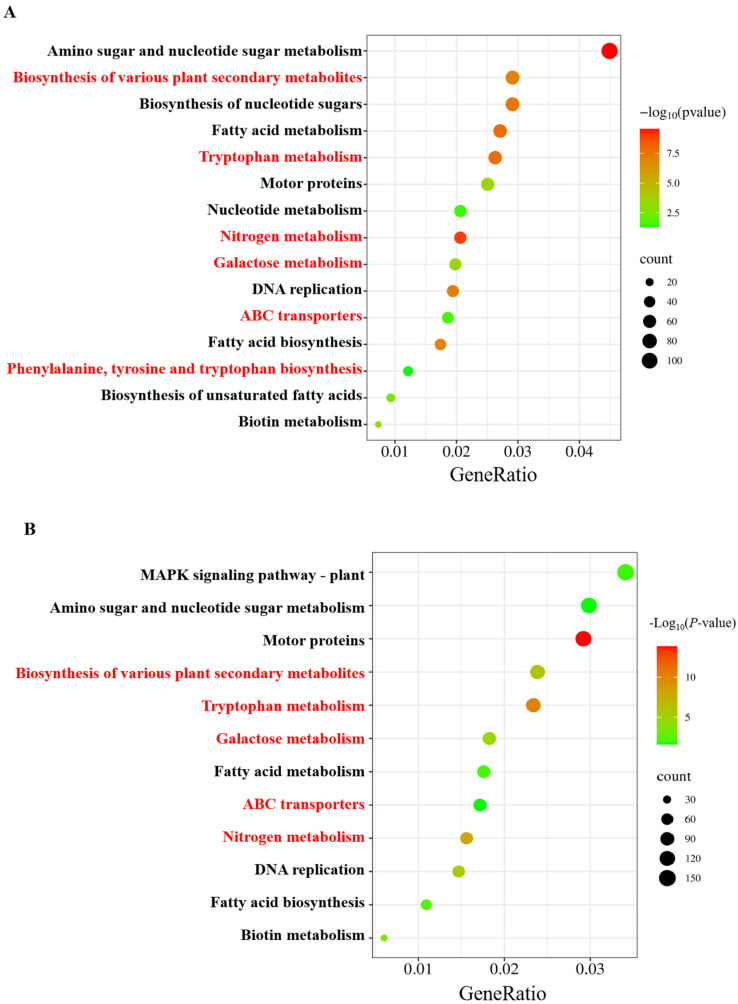
KEGG analyses of DEGs for the roots of wheat seedlings treated with 5 mM NO_3_^−^ (CK), 0.1 mM NO_3_^−^ (LN), and 0.1 mM NO_3_^−^ + 60 mg/L TIBA (LNT) for 48 h. (**A**) The KEGG enrichment analysis of LN compared with CK. (**B**) The KEGG enrichment analysis of LNT compared with LN. The *X*-axis in the figure is the GeneRatio; the *Y*-axis is the KEGG pathway. The size of the dots represents the number of genes annotated for the KEGG pathway, and the color gradient from red to green represents the significance level of the enrichment. The key enriched pathways for DEGs that were involved in auxin biosynthesis and transport and nitrogen metabolism were highlighted using the red font.

The KEGG analysis revealed that, in the LN vs. CK comparison, the pathways were mainly clustered into DNA replication, amino sugar and nucleotide sugar metabolism, nitrogen metabolism, tryptophan metabolism, ABC transporters, fatty acid metabolism, and phenylalanine, tyrosine, and tryptophan biosynthesis (Figure 5A). In LNT vs. LN, the significantly enriched pathways included the biosynthesis of various plant secondary metabolites, galactose metabolism, tryptophan metabolism, nitrogen metabolism, DNA replication, the MAPK signaling pathway in plants, and fatty acid metabolism (Figure 5B). These results indicate that a wide spectrum of physiological processes in wheat roots are affected by the LN and the presence of TIBA.

### 2.5. DEGs Related to IAA Synthesis, Oxidation, and Transport

**Figure 6:**
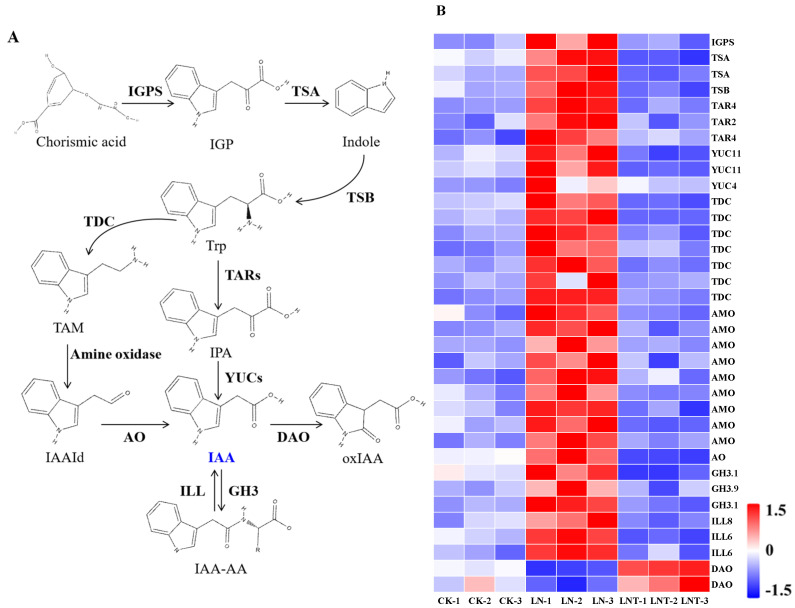
A schematic model of the Trp-dependent IAA biosynthetic pathway (**A**) and the expression of DEGs involved in the pathways (**B**). Wheat seedlings were treated with 5 mM NO_3_^−^ (CK), 0.1 mM NO_3_^−^ (LN), and 0.1 mM NO_3_^−^ + 60 mg/L TIBA (LNT) for 48 h. AO, indole-3-acetaldehyde oxidase; DAO, 2-oxoglutarate-dependent dioxygenase; GH3, indole-3-acetic acid-amido synthetase; IAA-AA, indole-3-acetic acid–amino acid conjugate; IAAId, indole-3-acetaldehyde; IGP, indole-3-glycerophosphate; IGPS, indole-3-glycerol phosphate synthase; ILL, IAA-amino acid hydrolase ILR1-like; IPA, indole-3-pyruvate; TAM, tryptamine; TAR, tryptophan aminotransferase-related protein; TDC1, tryptophan decarboxylase 1; Trp, tryptophan; TSA, tryptophan synthase alpha chain; YUCs, indole-3-pyruvate monooxygenase. Heatmap (**B**) showing the differential expression levels of the identified DEGs in the roots of wheat plants with different N treatments. The log_2_(FoldChange) values for individual genes are indicated by the color, as shown in the scale, with red indicating a high level of expression and blue indicating a low level of expression.

Considering that the GO terms and KEGG pathways were mainly clustered into tryptophan metabolism and biosynthesis, hormone binding, cell wall polysaccharide metabolism, and the cytoskeleton, and considering the importance of auxin in LR development, we next examined the expression of DEGs involved in the tryptophan-dependent auxin synthesis pathway. The transcriptomic analysis showed that the expression of auxin synthesis-related genes, including *AMO*, *AO*, *GH3*, *IGPS*, *ILL*, *TAM*, *TAR*, *TDC*, *TSA*, *TSB*, and *YUC*, was upregulated in the LN roots compared with CK and downregulated after the application of TIBA. Meanwhile, *DAO*, a dioxygenase for auxin oxidation catalyzing IAA oxidation, was downregulated in the LN roots and upregulated after the application of TIBA (Figure 6A,B). These results indicate that LN conditions promote the expression of auxin synthesis-related genes and the addition of TIBA mitigates these stimulatory effects.

**Figure 7:**
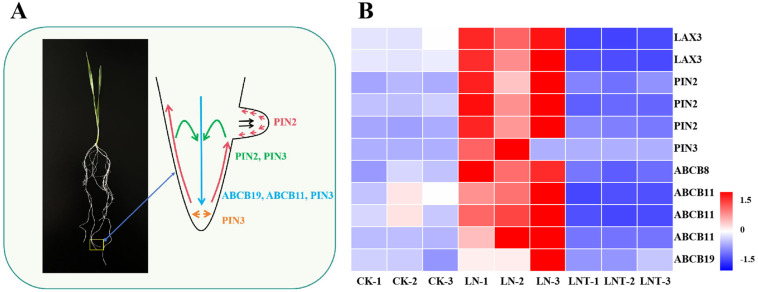
Schematic representation of major auxin transport types (**A**) and a list of genes related to auxin transport in wheat roots (**B**). The arrows in (**A**) indicate auxin flow mediated by a particular transporter. A heatmap (**B**) showing the differential expression levels of the identified DEGs in the roots with three treatments from three replicates. The color blocks represent log_2_-transformed fold changes, with red indicating a high level of expression and blue indicating a low level of expression.

Furthermore, we also analyzed the transcript abundance of genes involved in IAA transport processes. The results showed that the expression of genes encoding *LAX3*, PIN2, *PIN3*, *ABCB8*, *ABCB11*, and *ABCB19* was significantly upregulated under the LN conditions compared with the CK. After the addition of TIBA, the expression of these genes was significantly downregulated (Figure 7B). These data indicate that low NO_3_^−^ promotes while TIBA addition inhibits IAA transport. 

### 2.6. DEGs Involved in the Regulation of IAA Biosynthesis and Transport

Based on previous publications, we analyzed genes that were involved in the regulation of auxin synthesis and transport. These genes included the high-affinity nitrate transporter (*NRT*), auxin-responsive protein (*SAUR71*), PIN-LIKES (*PILS*), putative multidrug resistance protein (*MDR*), putative polyol transporter (*PLT*), ethylene-responsive transcription factor WRI1 (WRI1), agamous-like MADS-box protein AGL21 (*AGL21*), Fanconi anemia group J protein homolog (*BRIP1*), and transcription factor HY5. Among them, *AGL21*, *BRIP1*, *MDR*, *NRT2.1*, *PILS*, *PLT*, *SAUR71*, and *WRI1* were significantly upregulated and *HY5* was downregulated in LN vs. CK; conversely, *HY5* was upregulated and the others were significantly downregulated under LNT conditions compared with LN (Table 2).

The three squares in the heatmap from left to right represent wheat seedlings treated with 5 mM NO_3_^−^ (CK), 0.1 mM NO_3_^−^ (LN), and 0.1 mM NO_3_^−^ + 60 mg/L TIBA (LNT), respectively.

### 2.7. DEGs Related to Cell Wall Biological Properties

A total of 84 genes involved in cell wall modification were found to be differentially expressed, including those coding for cellulase, pectin acetylesterase, pectinesterase, pectate lyase, polygalacturonase, xyloglucan glycosyltransferase, probable xyloglucan glycosyltransferase, xyloglucan endotransglucosylase/hydrolase, endo-1,4-beta-xylanase, beta-1,4-xylosyltransferase, and beta-1,2-xylosyltransferase. The expression of all of these genes was significantly upregulated, while genes encoding the xylanase inhibitor protein were downregulated in the roots exposed to LN conditions compared with CK (Table 3). In the presence of TIBA, the expression of these DEGs was reversed compared with LN (Table 3). These data suggest that LN conditions may alter the root cell wall properties and that the genes encoding cell wall remodeling enzymes are repressed upon the addition of TIBA.

### 2.8. Expression of Genes Mediated by Auxin and Potentially Involved in Cell Wall Remodeling and LR Development

To explore the molecular mechanism by which auxin regulates cell wall loosening and LR initiation, we analyzed the transcript abundance of genes involved in cell wall remodeling and LR development. The results showed that the expression of genes encoding subtilisin-like protease (*AIR3*), plasma membrane ATPase (*H^+^-ATPase*), the glycosyl hydrolase family (*GLH*), NAC domain-containing protein (*NAC*), the nodulation signaling pathway (*NSP*), aquaporin (*PIPs*), and domain-containing protein RIC1 were significantly upregulated. After the addition of TIBA, the expression of these genes was significantly downregulated (Table 4).

### 2.9. DEGs Related to Cell Division, Elongation, and the Cell Cycle

**Figure 8:**
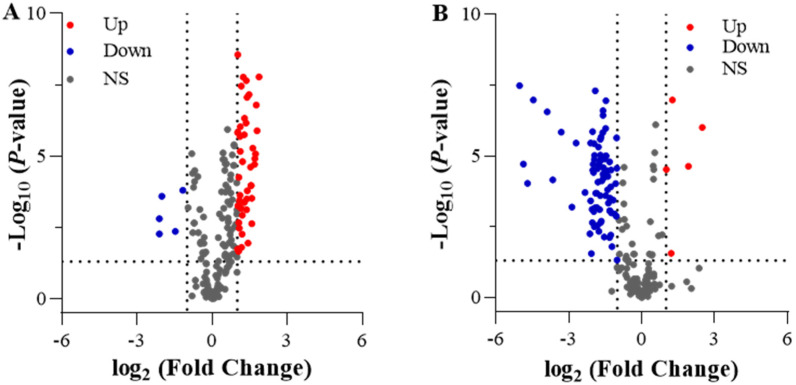
Volcano plots of DEGs involved in cell division, elongation, and the cell cycle. (**A**) LN vs. CK; (**B**) LNT vs. LN. Wheat seedlings grown in solution containing 5 mM NO_3_^–^ (CK), 0.1 mM NO_3_^–^ (LN), and 0.1 mM NO_3_^–^ + 60 mg/L TIBA (LNT) at 48 h after treatment. Red and blue dots indicate upregulated and downregulated DEGs, respectively, and gray dots indicate genes that were not differentially expressed.

**Figure 9:**
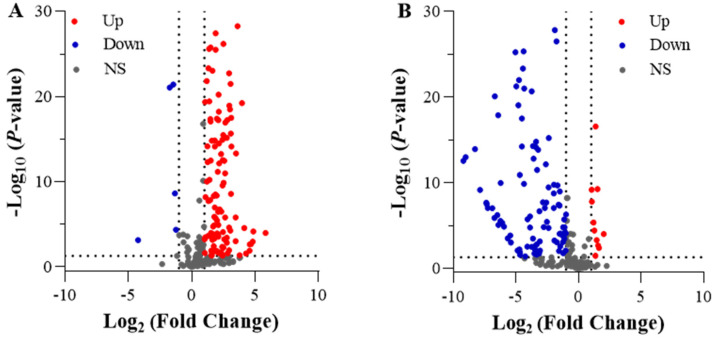
Volcano plots of DEGs encoding cell wall expansins. (**A**) LN vs. CK; (**B**) LNT vs. LN. Wheat seedlings grown in solution containing 5 mM NO_3_^–^ (CK), 0.1 mM NO_3_^–^ (LN), and 0.1 mM NO_3_^–^ + 60 mg/L TIBA (LNT) at 48 h after treatment. Red and blue dots indicate upregulated and downregulated DEGs, respectively, and gray dots indicate genes that were not differentially expressed.

A total of 203 genes were identified in all samples that were involved in cell division, elongation, and cell cycle processes. Specifically, the expression of 50 DEGs was upregulated and only 5 DEGs were downregulated in LN vs. CK (Figure 8A). In LNT vs. LN, the expression of 5 DEGs was significantly upregulated and 177 DEGs were markedly downregulated (Figure 8B). A total of 216 genes related to cell wall expansion were identified. In the comparison between LN and CK, 111 DEGs were significantly upregulated and only 5 DEGs were downregulated (Figure 9A), and, in LN vs. LNT, 11 were upregulated and 107 were downregulated (Figure 9B).

The majority of the DEGs encoding cell division control protein (*CDC*), the control of cell elongation transcription factor (*ILI*), microtubule-associated protein (*MAP*) involved in the cell cycle, expansins, and leucine-rich repeat extensin-like protein (*LRX*) were significantly upregulated in LN roots compared with CK roots, while most of these DEGs were downregulated in LNT roots compared with LN roots.

### 2.10. Verification of Hub DEGs via qRT-PCR

**Figure 10:**
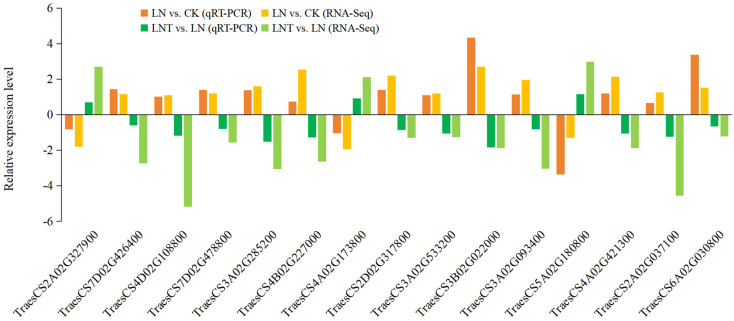
RNA-seq data accuracy verification. The column represents the 2^−ΔΔCt^ value of qRT-PCR analysis and log_2_(FoldChange) value of RNA-Seq. The values represent the mean ± SD. Positive values on the *Y*-axis indicate the upregulation of hub genes. Wheat seedlings grown in solution containing 5 mM NO_3_^–^ (CK), 0.1 mM NO_3_^–^ (LN), and 0.1 mM NO_3_^–^ + 60 mg/L TIBA (LNT) at 48 h after treatment.

qRT-PCR was performed to validate the transcriptome analysis. Fifteen key DEGs participating in auxin synthesis and transport, LR emergence, and cell wall relaxation were selected (Figure 10). The results showed that the expression levels of a total of 12 DEGs based on the RNA-seq analysis were upregulated in the roots of LN vs. CK (IAA-amino acid hydrolase, auxin efflux carrier, and xyloglucan endotransglycosylase/hydrolase protein genes), and three DEGs were significantly upregulated (2-oxoglutarate-dependent dioxygenase (*DAO*), xylanase inhibitor protein, and ethylene-responsive transcription factor). Meanwhile, in LNT vs. LN, these fifteen DEGs showed the reverse trend in their expression. Taken together, these results show that the differences in the expression levels of these candidate DGEs detected via RT-qPCR were in accordance with the RNA-seq data, which confirms the reliability of the RNA-seq results.

### 2.11. Root Tryptophan and IAA Content

**Figure 11:**
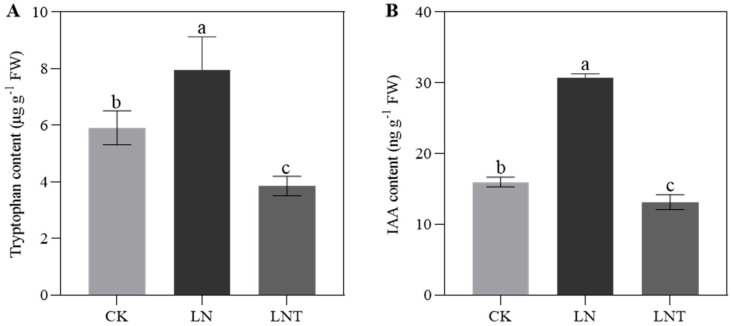
Content of root tryptophan (**A**) and IAA (**B**) in wheat seedlings grown under different N treatments. Different letters above columns indicate significant differences at *p* ≤ 0.05 between treatments. The values represent the mean ± SD of three replicates. Wheat seedlings grown in solution containing 5 mM NO_3_^–^ (CK), 0.1 mM NO_3_^–^ (LN), and 0.1 mM NO_3_^–^ + 60 mg/L TIBA (LNT) at 48 h after treatment.

To explore whether auxin is involved in LN-mediated LR formation, we determined the endogenous content of tryptophan and IAA. The results showed that LN stimulated increases in the content of root tryptophan of 34.26% and root IAA of 92.25% compared with the CK at 48 h after treatment (Figure 11A,B). The addition of TIBA reduced the IAA and tryptophan content by 57.21% (*p* < 0.05) and 51.42% (*p* < 0.05), respectively, compared with LN (Figure 11A,B). These data indicate that low NO_3_^–^ may promote and the addition of TIBA may inhibit IAA accumulation in the roots.

### 2.12. Effects of Different N Treatments on the Activity of Cell Wall Remodeling Enzymes and Cell Wall Composition in Wheat Roots

As shown in Table 5, the activity of pectinase, β-1,3-glucanase, polygalacturonase, exo-β-1,4-glucanase, cellulase, neutral xylanase, pectinate lyases, and pectinesterase was significantly higher in LN roots than in CK; LN also significantly increased these enzymes’ activity compared with LNT. Compared with the CK, LN significantly increased the galacturonic acid content and significantly decreased the content of cellulose and the degree of pectin-methyl esterification, while the opposite was true after TIBA addition compared with LN.

### 2.13. Fourier-Transform Infrared (FTIR) Spectra of Cell Wall Composition

**Figure 12:**
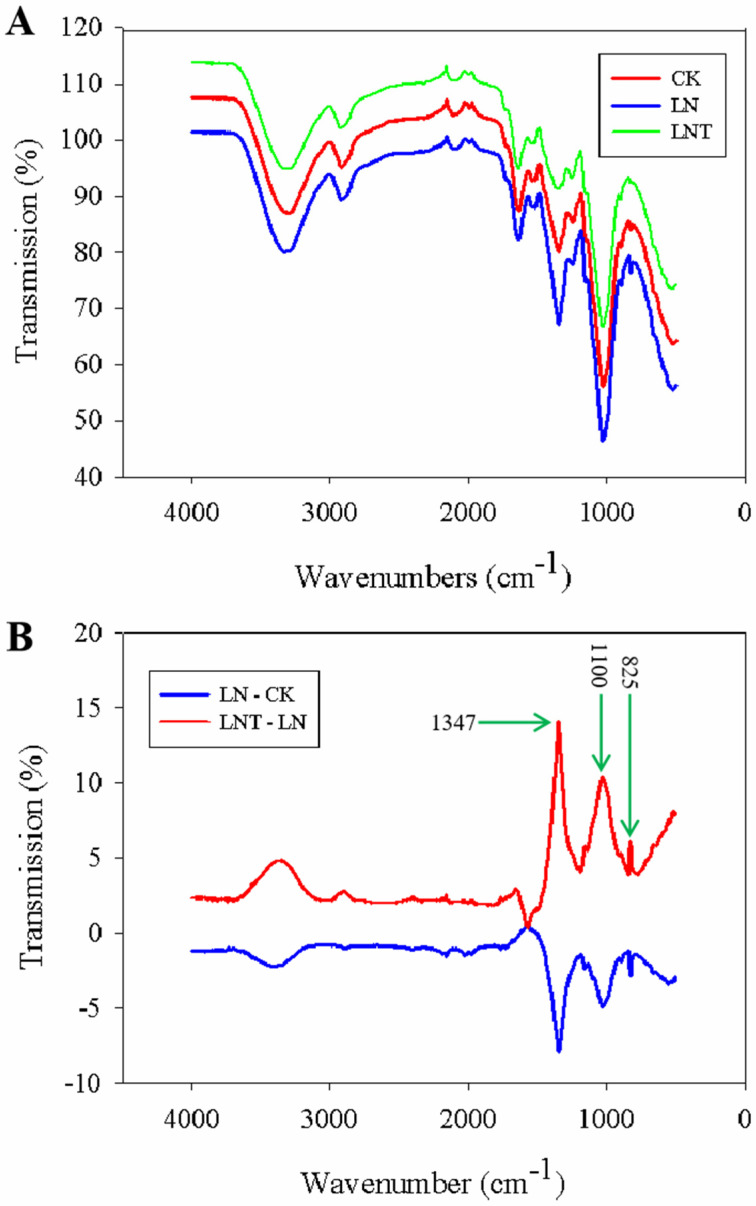
ATR-FTIR spectra obtained from the epidermal cell walls of LRP of wheat seedlings treated with 5 mM NO_3_^–^ (CK), 0.1 mM NO_3_^–^ (LN), and 0.1 mM NO_3_^–^ + 60 mg/L TIBA (LNT) for 48 h. (**A**) Difference spectra generated via the digital subtraction of the spectrum of the LN (0.1 mM NO_3_^–^) LRPs from that of the CK LRPs and the spectrum of the LNT (0.1 mM NO_3_^–^ + 60 mg/L) LRPs from that of the LN LRPs. (**B**) Graphs show the transmission (*Y*-axis) plotted against the wavenumber (*X*-axis).

To examine the effects of low NO_3_^–^ and TIBA on cell wall composition, FTIR spectra analysis was carried out. All samples were scanned at a wavenumber of 400 to 4000 cm^−1^ using FTIR. In this region, many absorption and vibration bands were identified. However, the FTIR spectra from the LRP cell walls of roots under different treatments showed similar profiles and were difficult to distinguish (Figure 12A). To facilitate the comparison, difference spectra were obtained for the cell wall components from the LN roots minus those from the CK roots, as well as spectra from the LNT roots minus the LN roots. Based on the difference spectra (Figure 12B), negative peaks at 825 cm^−1^, 1100 cm^−1^, and 1347 cm^−1^ caused by the LN conditions and positive peaks at these wavenumbers were observed after the addition of TIBA. These peaks can be attributed to D-(+)-galactose, D-(+)-glucose, and pectin (poly-galacturonic acid) (C-H vibrations), respectively [67,68]. The results of ATR-FTIR spectroscopy indicate that the polysaccharide components of the LRP cell wall decreased under low NO_3_^–^ conditions and increased after the addition of external TIBA (Figure 12B).

### 2.14. Cell Wall Plasticity

**Figure 13:**
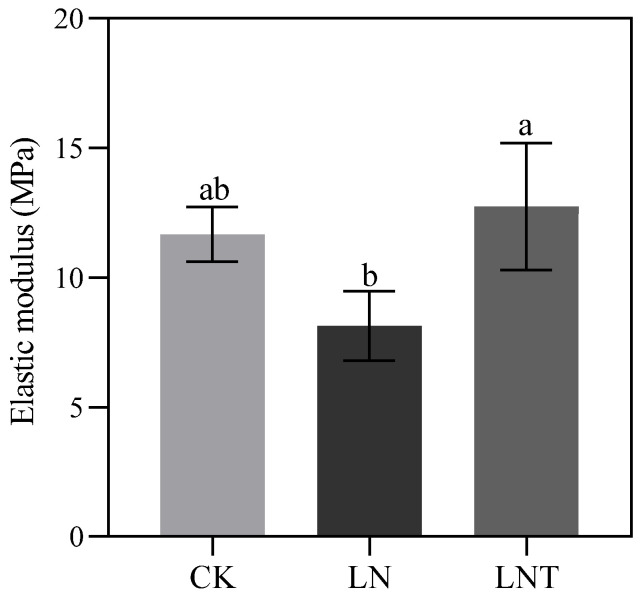
Graphs representing the LRP elastic moduli of wheat seedlings grown in 5 mM NO_3_^–^ (CK), 0.1 mM NO_3_^–^ (LN), or 0.1 mM NO_3_^–^ + 60 mg/L TIBA (LNT) at 48 h after treatment. Data are the mean ± SD from three LRPs. Different letters indicate statistically significant differences at *p* < 0.05.

The cell wall property of LRP was determined using atomic force microscopy (AFM). Figure 13 represents the average of the cell wall elastic modulus under different treatments. The results showed that the elastic modulus of LRP was greatly decreased in the LN roots compared with the CK and was significantly increased after the addition of TIBA (Figure 13), suggesting that the epidermal cell wall of LRP is more elastic under LN conditions than in CK and the addition of TIBA decreases the cell wall elasticity of LRP.

## 3. Discussion

### 3.1. Low NO_3_^−^ Promotes Lateral Root Occurrence and Increases Root Surface Area

Several studies have revealed that low NO_3_^−^ promotes LR growth [69,70,71]; however, the underlying mechanisms are not fully understood. In this study, we observed that LN increased the number of root tips and their surface areas (Figure 1B and Figure 2; Table 1). To examine whether the LN-induced changes in the root structure were related to auxin signaling, we applied TIBA, an auxin polar transport inhibitor, to evaluate LR development and root growth. The results showed that in the presence of TIBA, these parameters were significantly decreased. These results suggest that low NO_3_^–^ may induce LR formation and auxin signaling may be involved in this process. This view is consistent with the findings of Wang et al. (2021) [21]. 

### 3.2. Low NO_3_^–^ Promotes Root IAA Synthesis and Transport and Improves IAA Homeostasis

Tryptophan-dependent auxin synthesis is the canonical pathway [72,73]. In this pathway, IAA biosynthesis is initiated from tryptophan, which is produced from indole-3-glycerophosphate (*IGP*) in the presence of tryptophan synthase (*TSA*) [74]. Tryptophan aminotransferases catalyze the conversion of tryptophan to indole-3-pyruvate (*IPA*), which is converted to auxin by indole-3-pyruvate monooxygenases (*YUCs*) [75,76]. Alternatively, tryptophan decarboxylases (*TDCs*) catalyze tryptophan to trigger the N-hydroxylation of tryptamine (*TAM*). TAM is further converted to indole-3-acetaldehyde (*IAAId*), a process requiring the participation of amine oxidase [77]. IAAId is then catalyzed to generate auxin via indole-3-acetaldehyde oxidase (*AO*) [78]. In the current study, we observed that the expression of genes encoding auxin synthesis-related enzymes was significantly upregulated under LN conditions while being decreased in the presence of TIBA (Figure 6B). In addition, low NO_3_^−^ improved auxin homeostasis by downregulating the expression of *DAO*, which oxidizes IAA into oxIAA [79], and by upregulating the expression of *GH3* and *ILL*; both genes are responsible for IAA-amido conjugation and IAA-AA hydrolyzation [80,81,82]. Meanwhile, the addition of TIBA caused the inactivation of IAA to oxIAA via *DAO* and inhibited the activation of IAA from IAA-AA conjugates due to the decreased expression of *ILL* (Figure 6). Physiological determination also revealed increases in tryptophan and IAA content under LN conditions (Figure 11A,B) and decreases after the addition of TIBA. These results indicate that a low level of NO_3_^–^ promotes auxin biosynthesis and improves IAA homeostasis in the roots of wheat seedlings, and, unexpectedly, TIBA may inhibit root auxin accumulation, partially due to a decrease in IAA biosynthesis and an increase in IAA degradation. 

Polar transport plays a pivotal role in improving auxin homeostasis. Multidrug resistance (*MDR*), P-glycoprotein (*PGP*), ATP binding cassette B (*ABCB*) is a member of the ABC protein family [40] and plays crucial roles in the control of key developmental events in plants [83,84]. *OsABCB8* is localized in the cortex of the epidermis of the root maturation zone [85] and is responsible for auxin transport. *ABCB11* acts in concert with *ABCB1* and *ABCB19* (initially named *AtPGP1* and *AtMDR1*) in long-distance transport [86] and is specifically associated with auxin transport [87]. The free IAA content is severely reduced in *AtABCB1* and *AtABCB19* mutant hypocotyls and roots and even more drastically reduced in the *AtABCB1 AtABCB19* double mutant [88]. In addition, *NRT2.1* plays a positive role in regulating LR development by affecting auxin polar transport under low NO_3_^–^ conditions [35]. As shown in Figure 7B, all genes associated with auxin transport were upregulated under low NO_3_^–^ conditions and downregulated after the addition of TIBA. Therefore, we can deduce that low NO_3_^–^ may improve auxin polar transport, which plays a pivotal role in root auxin homeostasis and LR formation. Similarly, in maize, low NO_3_^–^ positively affects LR formation depending on more auxin accumulation in LR primordia while high NO_3_^–^ inhibits root growth due to reduced IAA levels in the roots [89]. In rice, low nitrogen significantly increases root length and LR count, largely by promoting the polar transport of auxin from the shoot to the root and by inhibiting the expression of genes that negatively regulate ethylene and jasmonic-acid-related pathways [90]. In cotton (*Gossypium hirsutum* L.), a moderate level of nitrogen increased lateral root growth by significantly decreasing abscisic acid and salicylic acid contents compared with high nitrogen concentration [91]. Taken together, crosstalk among phytohormones may play an essential role in low nitrate-induced LR development. 

### 3.3. DEGs Involved in the Regulation of IAA Biosynthesis, Transport, and Signaling

Finely balanced auxin homeostasis is dependent on numerous factors such as genes encoding *PLT*, *WRI1*, *AGL21*, *BRIP1/2*, *BZR1*, *GRE6*, *IDD*, *WAG1*, and *HAT* (Table 2). Auxin has been proposed as a long-range signal from shoots to roots in response to nitrate [92], and interactions between auxin and nitrate signaling cooperatively regulate LR development [4,93]. In this study, we found that the expression of genes encoding *PLT*, *WRI*1, *AGL21*, B*RIP1/2*, *BZR1*, *GRE6*, *IDD*, *WAG1*, and *HAT* was upregulated in the roots exposed to LN and downregulated in the presence of TIBA, while *HY5* was downregulated under low NO_3_^–^ conditions and upregulated after the addition of TIBA (Table 2). Hence, we conjecture that a large number of factors are involved in low-NO_3_^–^-mediated auxin biosynthesis and transport. 

### 3.4. Low NO_3_^–^ Modulates Cell Wall Remodeling and Elasticity

The plant cell wall is a natural nanostructure composed of a variety of biological macromolecules, such as cellulose, pectin, hemicellulose, lignin, and a small amount of protein [94], and is differently modified in structure and composition by the activity of a wide range of enzymes [95]. The cell wall can also control plant cell expansion via a finely regulated balance between the flexibility and structural integrity of the cell [96,97]. The primary cell wall remodeling enzymes/proteins include POLYGALACTURONASE (PG), EXPANSIN (EXP), and GLH [41,57]. PG is involved in the degradation of demethylated pectin in the cell wall. *EXP17* is another component of the plant cell wall that lacks enzymatic activity but can nevertheless relax the plant cell wall [62]. The role of auxin in cell expansion is linked directly to cell wall modification [98]

Pectin, a homogalacturonan, consists of α-1,4-linked galacturonic acids. Homogalacturonans are secreted in a highly methylesterified form and are selectively de-methylesterified by pectin methylesterases (PMEs) [99]. PMEs with the resulting low DPM can facilitate cell wall loosening and cell expansion and thus tissue elongation [100,101]. Polygalacturonase can hydrolyze pectin to produce galacturonic acid, facilitate root cell wall remodeling, promote LRP cell division and LR elongation, and ultimately promote LR formation [102]. Cellulose and hemicellulose are the main components of the cell wall and are degraded via the action of cellulases [103]. Xyloglucan, β-glucan, and arabinoxylan form a homogeneous architecture of hemicellulose. In the epidermal cells overlying the LRP, the cross-linking xyloglucan can be modified primarily via the action of xyloglucan endotransglucosylase/hydrolase (XTH), polygalacturonase, and expansins and thereby facilitate the separation of microfibrils upon cell wall acidification [104]. 

Expansins (EXPs), a family of non-enzymatic proteins with cell wall loosening activity [105], can relieve the tension of the cell wall and loosen the cell wall by disrupting the hydrogen bonds between the cellulose microfibrils and the matrix polysaccharides [106]. EXP family genes, such as EXPANSIN A1 (*EXPA1*), *EXP14*, and *EXP17*, have also been reported to promote LR formation in response to auxin [107,108]. In this study, we found that the expression of most genes related to cell wall remolding enzymes/proteins, including cellulase, polygalacturonase, pectinesterase, XTHs, PMEs, and EXPs, were upregulated in the roots of wheat seedlings when exposed to LN (Figure 8B and Figure 9; Table 3) and downregulated by the addition of TIBA (Figure 9). Our physiological assays revealed that the activity of pectinase, polygalacturonase, β-1,3-glucanase, exo-β-1,4-glucanase, cellulase, neutral xylanase, and pectinate lyases was significantly increased under LN conditions, while the addition of TIBA led to significant decreases in the activity of these enzymes (Table 5). As a consequence, the content of cellulose and the degree of pectin-methyl esterification were significantly decreased and the galacturonic acid content was enhanced significantly in LN roots compared with CK, while the addition of TIBA reversed these changes (Table 5).

To further verify whether LN treatment could induce cell wall relaxation in the roots, we determined the cell wall properties of LRP using FTIR and AFM analysis, and the results showed that LN decreased the number of polysaccharide macromolecules (Figure 12B) and increased the cell wall elasticity of the LRP epidermis (Figure 13). Adding TIBA increased the number of polysaccharide macromolecules in the LRP and increased cell wall stiffness (Figure 12B and Figure 13).

Overall, these results indicate that low NO_3_^–^ positively regulates the pathway of cell wall remodeling in the roots as indicated in Figure 4, which may result in higher cell wall extensibility and plasticity and promote cell expansion and elongation during LR formation. These processes are largely mediated by auxin signaling.

### 3.5. Regulation of Low-NO_3_^–^-Induced Cell Wall Elasticity and LR Formation via Auxin Signaling

Plasma-membrane-bound ATPases transport protons from the protoplast into the apoplast and consequently acidify the extracellular matrix. This acidification, in turn, activates expansins and other cell wall remodeling enzymes, loosens the network of cellulose and additional cell wall components, and promotes cell expansion and shoot and root growth in plants [109,110]. These processes are mediated by auxin [29]. In the present study, a transcriptome-wide analysis showed that the expression of genes encoding plasma membrane ATPase 10 was upregulated under low NO_3_^–^ conditions and downregulated after the addition of external TIBA.

Aquaporins (AQPs) are membrane channels that facilitate water movement across cell membranes [111,112]. It has been hypothesized that AQPs contribute to maintaining adequate cell turgor pressure by allowing water influx across the plasma membrane and the tonoplast [61,113,114]. Péret et al. (2012) [61] demonstrated that auxin promoted water transfer from the overlying cells into the primordium by regulating AQPs and thus improved tissue hydraulics and promoted LR emergence. In our study, the transcript expression of aquaporin genes was significantly upregulated under LN conditions compared with CK, while it was downregulated after the addition of TIBA.

PR1 homolog PRH1 regulates the expression of *EXP* genes and affects cell wall loosening during LR development [115,116]. The expression of *PRH1* was induced upon auxin treatment, and mutants of *PRH1* had fewer LRs [117]. In the present study, the expression of the *PRH1* and *EXP* genes was upregulated under low NO_3_^–^ conditions and downregulated after the addition of TIBA (Figure 9 and Table 3), further indicating that auxin signaling may be involved in low-NO_3_^–^-induced LR formation.

*NAC1*, a new member of the NAC family, is induced by auxin, activates the expression of auxin-responsive gene *AIR3*, and mediates auxin signaling to promote LR development by weakening the connections among cells during root development in *Arabidopsis thaliana* [57,118]. In our study, the transcript expression of the *NAC1* and *AIR3* genes was significantly upregulated under LN conditions compared with CK, while it was downregulated after the addition of TIBA (Table 4), indicating that auxin, *NAC1*, and *AIR3* are involved in LR formation. This view is supported by the finding that *NAC1* functions as an intermediate between auxin and *AIR3*-mediated LR development [57,118].

The auxin influx carrier *LAX3*, which is specifically expressed near the LRP, modulates cell wall remodeling enzymes/proteins including PG, GLH, AIR3 (a subtilisin-like protease), and EXPs [57,62]. In this study, the DEGs coding for LAX3, PG, GLHs, AIR3, and EXPs were upregulated by low NO_3_^–^ and downregulated after the addition of TIBA (Figure 7B and Figure 10; Table 2 and Table 3). The expression of small auxin-upregulated RNAs (SAURs) is rapidly activated by auxin [119]. The auxin-induced expression of *SAUR71* in the steles of young roots and hypocotyls promotes cell growth and organ elongation and thus positively regulates seedling growth [120]. Receptor-like protein kinase FERONIA interacts with RIC1 and is involved in auxin signaling [64,121], probably affecting auxin polar transport and distribution [122,123]. The *PUCHI* gene, encoding an APETALA2-like transcription factor, was regulated by auxin in the definition of primordium boundaries during LR outgrowth [124]. In the present study, the transcriptome-wide analysis showed that the expression of genes encoding SAUR71, APETALA2-like protein, and RIC1 was upregulated under low NO_3_^–^ conditions and downregulated after the addition of external TIBA (Figure 6B and Table 2).

Based on these results, we conclude that the LN conditions enhanced cell wall elasticity and LR formation by regulating the cell wall remodeling pathway, and a large set of transducers downstream of auxin signals may be involved in these processes. 

### 3.6. Low NO_3_^–^ Promotes Root Cell Division and Elongation

In addition to cell wall expansion and cell wall modification, root morphogenesis is also regulated by cell division and elongation [71,125,126,127]. It has been reported that auxin promotes LR initiation by enhancing cell cycle activity and pericycle activation at the G1-to-S transition [128,129]. In addition, during LR morphogenesis, these processes are mediated by microtubule remodeling, which is in turn modulated by auxin [130]. TETRATRICOPEPTIDE-REPEAT THIOREDOXIN-LIKE 1 (TTL1) is also related to the microtubule structure and is involved in regulating the cell wall elasticity of root rhizodermal cells in the elongation zone [59]. BRs induce the expression of cyclin D genes, which promote cell division and play important roles in LR initiation and elongation [58]. In this study, most DEGs involved in cell division, elongation, and the cell cycle, such as *CYCD6*, *CDC, ILI, MAP, TTL1,* and *BZR1,* were upregulated in response to low NO_3_^–^ conditions compared with CK and downregulated with TIBA addition (Figure 8A). Therefore, we speculate that low NO_3_^–^ conditions can promote root cell proliferation and cell elongation and thus boost LR formation. 

## 4. Materials and Methods

### 4.1. Plant Materials and Plant Culture

Seeds of wheat (cultivar Jimai 22) were surface-sterilized using 70% ethanol for 40 s and washed 4 times with distilled H_2_O. The sterilized seeds were placed in Petri dishes, covered with moist filter paper above and below, and germinated in Petri dishes at 24 °C. After 3 days, 15 seedlings of the same size were selected and fixed in each plastic pot (10 cm × 8 cm × 5 cm in length, width, and height) containing 300 mL distilled H_2_O and then placed in a light growth chamber at 24 °C (light)/20 °C (dark) for 14 h/10 h. The light intensity was 450 μmolm^−2^ s^−1^, and the relative humidity was maintained at 70 ± 5%. The 3-day-old seedlings were grown in distilled water for 5 d to exhaust the seed reserves. After this, the 8-day-old wheat seedlings were treated with 1/2 × Hoagland’s nutrient solution. In our preliminary experiments, we found that the roots of the 8-day-old seedlings grown in 1/2 strength Hoagland’s nutrient solution containing 5 mM NO_3_^–^ grew best and that 0.1 mM NO_3_^–^ could significantly promote LR development. Therefore, the following experiments were conducted using 8-day-old seedlings that were hydroponically cultured in 1/2 strength Hoagland’s nutrient solution containing 5 mM NO_3_^–^ (CK; applied as Ca(NO_3_)_2_ at 2.5 mM), 0.1 mM NO_3_^–^ (LN; applied as Ca(NO_3_)_2_ at 0.05 mM), or 0.1 mM NO_3_^–^ + 60 mg/L TIBA (LNT). The solution was renewed every day. Each treatment was repeated in triplicate. CaCl_2_ was supplemented with the LN and LNT nutrient solution to maintain the same concentration of Ca^2+^ as in the CK solution. The nutrient solution was renewed every day.

### 4.2. Biomass Determination

To determine the biomass, the wheat seedlings were collected at 0, 12, 24, and 48 h after treatment and dried with absorbent paper. The shoots and roots were separated and then oven-dried at 60 °C for 30 min and then at 105 °C to a constant weight. The dried samples were weighed with a four-digit balance. Three biologically independent experiments, each with three replicates and 15 plants per replicate, were performed to calculate the fresh weight. The net increase was calculated by subtracting the dry weight of shoots or roots collected at 0 h from that measured 12, 24, or 48 h after treatment.

### 4.3. Root Architecture Measurements

At 48 h after treatment, intact root systems were spread out in a Perspex tray (200 mm × 250 mm) containing distilled H_2_O to untangle the roots and minimize root overlap, scanned using a scanner (Epson 1680, Seiko Epson Corporation, Nagano, Japan) at a resolution of 300 dpi, and analyzed using a WinRHIZO root analyzer (WinRHIZO, Régent Instruments Inc., Montreal, QC, Canada) to obtain the total root length, the root surface area, and the number of root tips. Nine roots with a uniform size were selected for each treatment.

### 4.4. Transcriptome Sequencing

#### 4.4.1. RNA Extraction and Detection

Wheat roots were harvested at 48 h after treatment and stored at −80 °C, and the total RNA was extracted and subjected to transcriptome sequencing. Total RNA was extracted from the roots using TRIzol (Invitrogen, Beverly, MA, USA) and treated with RNase-free DNase I to remove residual DNA. Absorbance ratios A260/A280 and A260/A230 were detected using a NanoPhotometer spectrophotometer to determine the RNA purity, and RNA integrity was accurately measured using an Agilent 2100 (Agilent, Palo Alto, CA, USA) bioanalyzer. The RNA extracted from all samples was complete, with A260/A280 ratios between 1.8 and 2.1 and an A260/A230 ratio greater than 1.8. Lastly, library quality was assessed on the Agilent Bioanalyzer 2100 system.

#### 4.4.2. Library Construction and Quality Inspection

Total RNA was used as the input material for RNA sample preparation. Briefly, mRNA was purified from total RNA using poly-T oligo-attached magnetic beads. First-strand cDNA was synthesized using a random hexamer primer and M-MuLV Reverse Transcriptase (RNase H-). Second-strand cDNA synthesis was subsequently performed using DNA Polymerase I and RNase H. The remaining overhangs were converted into blunt ends via exonuclease/polymerase activity. After the adenylation of the 3′ ends of the DNA fragments, adaptors with a hairpin loop structure were ligated to prepare for hybridization. In order to select cDNA fragments of preferentially 370–420 bp in length, the library fragments were purified with the AMPure XP system (Beckman Coulter, Beverly, CA, USA). Then, PCR was performed with Phusion High-Fidelity DNA polymerase (NEB, Ipswich, MA, USA), universal PCR primers, and the Index (X) Primer.

After the library was constructed, a Qubit 2.0 Fluorometer (Thermo Fisher Scientific, Waltham, MA, USA) was used for preliminary quantification, diluting the library to 1.5 ng/μL, and then the Agilent 2100 bioanalyzer was used to detect the insert size of the library. Once the insert size met the expectations, qRT-PCR was used to accurately quantify the effective concentration of the library (the effective concentration of the library was higher than 1.5 nM) to ensure its quality.

#### 4.4.3. Sequencing

The sequencing platform was the Illumina NovaSeq 6000 (Illumina, San Diego, CA, USA). After the library inspection was completed, different libraries were pooled according to the requirements for the effective concentration and target offline data volume for Illumina sequencing. Four fluorescently labeled dNTP, DNA polymerase, and adaptor primers were amplified to the sequenced flow cells. When each sequencing cluster extended the complementary strand, each fluorescently labeled dNTP was released. The sequencer captured the fluorescence signal and converted the light signal into the sequencing peak through computer software to obtain the sequence information of the fragments to be measured.

#### 4.4.4. Data Quality Control

To ensure the quality and reliability of the data analysis, raw data (raw reads) in fastq format were first processed through fastp software v0.20.1. In this step, clean data (clean reads) were obtained by removing reads containing adaptors, reads containing poly-N, and low-quality reads from the raw data. At the same time, the Q20, Q30, and GC content of the clean data were calculated. All downstream analyses were based on clean data of high quality.

#### 4.4.5. Analysis of Total Differentially Expressed Genes (DEGs)

After the quantification of gene expression, the gene expression data were statistically analyzed and genes with significantly different expression levels in different states were screened. The original read count was first standardized (normalization) and mainly corrected for the review sequencing depth. The statistical model was then used to calculate the hypothesis testing probability (*P*adj) and perform multiple hypothesis testing correction to obtain the FDR value (false discovery rate; *P*adj is its common form). Finally, the number of DEGs for each comparative combination was counted and screened to analyze the expression of the target genes. The screening criteria were |log_2_(Fold Change)| ≥ 1, *p* ≤ 0.05.

#### 4.4.6. GO Term and KEGG Enrichment Analysis

Gene Ontology (GO; http://www.geneontology.org; accessed on 1 April 2024) is the international standard classification system for gene function. The R software’s (R Core Team, 2021, version 4.4) clusterProfiler package version 3.19 was used to analyze the GO enrichment of the DEGs and genes, and the corresponding GO number was obtained via the ID correspondence or sequence annotation method. Then, the GO distribution related to the DEGs was obtained across three levels: biological process (BP), molecular function (MF), and cellular component (CC). GO terms with corrected *p*-values less than 0.05 were considered significantly enriched by differentially expressed genes. KEGG (http://www.genome.jp/kegg; accessed on 1 April 2024) is a database resource for the understanding of high-level functions and utilities of the biological system from molecular-level information. Blast2GO software version 6.0 was used to test the statistical enrichment of the differentially expressed genes in the KEGG pathways (corrected *p-*value < 0.05).

### 4.5. RNA Isolation and Reverse Transcription Quantitative Real-Time Polymerase Chain Reaction (qRT-PCR)

The total RNA for qRT-PCR was isolated from wheat roots using Trizol reagent (Invitrogen, Carlsbad, CA, USA) according to the manufacturer’s instructions. All qRT-PCR reactions were performed in 96-well PCR plates (ABgene, Portsmouth, NH, USA). The samples were processed using the same reagents and protocol described for wheat tissue. The quantitative qRT-PCR experiments were performed in a LightCycler^®^ 480 thermocycler (Roche, Burlington, NJ, USA). The qRT-PCR conditions were as follows: denaturing at 94 °C for 30 s; 45 cycles of 94 °C for 5 s, 60 °C for 15 s, and 72 °C for 10 s, with melting and cooling. The transcript levels were normalized by applying the 2^−ΔΔCt^ method. Actin was used as an internal standard to normalize the differences in the template amounts. Three biological replicates were performed. Differences between the two samples were assessed using the Student’s *t*-test method, and *p* < 0.05 was considered to indicate a significant difference in content.

### 4.6. 2,3,5-Triodobenzoic Acid (TIBA) Treatment

To confirm the role of auxin in regulating LR development under low NO_3_^−^ conditions, TIBA, an inhibitor of auxin polar transport, was dissolved in dimethyl sulfoxide and added to the 0.1 mM NO_3_^−^ solution at a final concentration of 60 mg/L (Sigma-Aldrich, St. Louis, MO, USA) to determine root phenotypic changes.

### 4.7. Determination of Tryptophan, IAA, and Cell Wall Components

To determine the content of tryptophan, free auxin, and cell wall components, the roots were collected at 48 h after treatment, washed five times with distilled water, and stored in liquid nitrogen immediately to prevent the enzymatic and thermal degradation of these compounds.

Roots (approximately 0.1 g) were collected at 48 h after treatment, ground immediately to a fine powder in liquid nitrogen, and transferred to a 1.5 mL tube, to which 20 µL of the solution of internal standards (hexyl hydride) was added. Subsequently, 400 µL of hexane was added to each sample and they were vortexed, filtered through 0.45 μm pore size filters, and stored. The tryptophan was quantified using high-performance liquid chromatography (HPLC; Rigol L3000, RIGOL, Beijing, China). HPLC was performed using a Sepax C18 column (50 mm × 4.6 mm, 5 μm; Sepax, Beijing, China) with a column temperature of 40 °C. The mobile phase comprised solvent A (0.76% [*v*/*v*] sodium acetate) and solvent B (80% [*v*/*v*] methyl cyanide), the flow rate was 1.0 mL min^−1^, and the injection volume was 10 µL. 

IAA was quantified via HPLC (Rigol L3000, RIGOL, Beijing, China) with a UV detector (254 nm). Briefly, fresh roots (approximately 0.1 g) were homogenized, and the free IAA was extracted using an aqueous solution of methanol. The extracts were purified through a C18 column on a solid-phase extractor, and the eluate was collected. The IAA was eluted with 80% methanol, and the eluent was evaporated to dryness under a stream of nitrogen. The operating conditions included a Sepax-C18 column (4.6 mm × 250 mm, 5 μm) and a mobile phase of methanol–water (2:3; *v*/*v*); the excitation and emission wavelengths were 275 nm and 345 nm, with a flow rate of 0.8 mL min^−1^, an injection volume of 10 µL, and a column temperature of 35 °C.

The cellulose content was determined using a cellulose content test kit (Suzhou Keming Biotechnology Co., Ltd., Suzhou, China). The samples were dried to a constant weight at 80 °C, pulverized, and passed through a 40-mesh sieve. Then, 0.01 g of sample was weighed, and the cell walls in the samples were extracted first with 80% ethanol and acetone, and then cellulose was extracted with concentrated sulfuric acid and distilled water, after which the supernatant was collected. The supernatant was mixed with the corresponding reagent in the assay kit and the experimental steps were carried out according to the instructions. The absorbance of each sample was measured at 620 nm using a NanoPhotometer P-Class USB ultraviolet spectrophotometer (Shimadzu UV-2600, Tokyo, Japan). All assays were performed using three biological replicates.

The content of root galacturonic acid was detected with a galacturonic acid content kit (Suzhou Keming Biotechnology Co., Ltd., Suzhou, China). Fresh roots (approximately 0.1 g) were homogenized using a mortar and pestle with 1 mL of pre-chilled extraction solution (80% methanol *v*/*v*, 100 mM imidazole, pH 7.0) and then heated at 90 °C for 2 h. The homogenate was transferred into 2 mL Eppendorf tubes and centrifuged at 8000× *g* at 4 °C for 10 min, and then the supernatant was collected. Next, the supernatant was mixed with the corresponding reagent in the assay kit and the experimental steps were carried out according to the instructions. The spectrophotometric value of the solution was measured using a spectrophotometer (UV-2600, Shimadzu, Tokyo, Japan). The solution was measured at 530 nm to obtain the absorbance values. The galacturonic acid content was calculated according to the manufacturer’s instructions.

### 4.8. Activity of Enzymes and Pectin Methylation Degree Involved in Cell Wall Remodeling

The pectin methylation degree of the samples was determined using a pectin methylation degree assay kit (Suzhou Keming Biotechnology Co., Ltd., Suzhou, China). Briefly, the roots (0.1 g) and 1 mL ethanol (95%) were ground to a powder in a pre-cooled mortar. After incubation in an ice bath for 20 min, the tubes were centrifuged at 4 °C and 10,000 × *g* for 10 min. The supernatant was discarded and distilled water was added; then, the sample was centrifuged at 10,000× *g*, 4 °C for 10 min, and the resulting supernatant was discarded again and 1 mL of phosphate buffer was added (2 mM ethylene diamine tetraacetic acid, 4% polyvinyl pyrrolidone, 1 M NaCl). The sample was then incubated in a water bath at 90 °C for 2 h and cooled to room temperature. The extract was centrifuged at 4 °C at 10,000× *g* for 10 min, and then the supernatant was collected. Next, the supernatant was mixed with the corresponding reagent in the assay kit and the experimental steps were carried out according to the instructions (Suzhou Keming Biotechnology Co., Ltd., Suzhou, China). Finally, after incubation at 25 °C for 20 min, the absorbance of the resulting solution was measured at 550 nm and 530 nm using a spectrophotometer (Shimadzu UV-2600, Shimadzu, Tokyo, Japan). Pectin methylation degree (%) = methanol content/galacturonic acid content × 100%.

The pectinesterase, pectinate lyase, pectinase, neutral xylanase, ploygalacturonase, β-1,3-glucanase, β-1,4-glucanase, and cellulase activity was determined according to the instructions of the reagent kit (Suzhou Keming Biotechnology Co., Ltd., Suzhou, China). Briefly, root tissue (0.1 g) was homogenized at 4℃ in 1 mL of extraction buffer (1 mM ascorbic acid, 3 mM β-mercaptoethanol, 0.5 mM phenylmethanesulfonylfluoride or phenylmethylsulfonyl fluoride, 2% polyvinyl pyrrolidone, and 1 mM ethylene diamine tetraacetic acid; pH 7.8) and centrifuged at 8000× *g*, 4 °C for 15 min, respectively. The supernatant was collected via enzyme extraction. Then, the supernatant was mixed with the corresponding reagent in the assay kit and the experimental steps were carried out according to the instructions (Suzhou Keming Biotechnology Co., Ltd., Suzhou, China). The absorbance of each sample was measured at 235 nm (pectinate lyases), 540 nm (pectinase, neutral xylanase, polygalacturonase, β-1,4-glucanase), 550 nm (pectinesterase, β-1,3-glucanase), and 620 nm (cellulase) using a spectrophotometer (Shimadzu UV-2600, Tokyo, Japan). Enzymatic activity was calculated according to the manufacturer’s instructions. All assays were performed using three biological replicates.

### 4.9. Atomic Force Microscopy

For AFM imaging, root blocks were fixed onto a glass slide using nail polish and submerged under a drop of distilled H_2_O at room temperature during imaging. A silicon nitride cantilever (ScanAsyst-Fluid, Bruker, Madison, WI, USA) with a nominal spring constant of 0.7 N/m (frequency = 150 kHz) and a nominal tip radius of 20 nm was used. Cell wall elasticity was measured using an indentation depth of 150 to 250 nm and an indentation force of 30 nN, and at least three indentation positions were chosen for each LRP. The quantification of elasticity (elastic modulus) was achieved with a commercial AFM BioScope Resolve (Bruker) under PeakForce Quantitative Nanoscale Mechanical (QNM) mode at 64 × 64 pixels. The AFM data were processed and analyzed with the NanoScope Analysis software (Bruker, version 1.8). At least five LRPs from three seedlings of each treatment were collected to determine the elastic modulus.

### 4.10. FTIR Analysis

FTIR analysis was performed using attenuated total reflectance Fourier-transform infrared spectroscopy (ATR-FTIR) (Nicolet iS50, ThermoNicolet, Madison, WI, USA,). The LRPs were cut and dried. ATR-FTIR images were obtained with a mid-IR spectral device at a 4 cm^−1^ resolution using a Spotlight 400 FTIR (Perkin Elmer, Waltham, MA, USA). According to the manufacturer’s procedure, the LRP was pushed via pressure into direct contact with the tip of the plane diamond. A total of 64 scans per pixel were performed in order to enhance the signal-to-noise ratio. The spectrum was then corrected using spectral image software (v.1.6.4) and the empty scan was used as the background. The ATR-FTIR spectra were obtained from nine root samples for each treatment.

### 4.11. Statistical Analysis

All data were statistically analyzed via analysis of variance (ANOVA) using the SPSS software package (SPSS 27.0.1; SPSS Inc., Chicago, IL, USA). Duncan’s multiple range test was performed at *p* ≤ 0.05 on each of the significant variables measured. All values were expressed as means ± standard deviations (SD).

## 5. Conclusions

In summary, our results indicate that a low concentration of nitrate can promote the epidermal cell wall relaxation of lateral root primordia and thus lateral root development, during which root auxin signaling plays an essential role. This study provides useful information on the interactions between low nitrogen conditions and auxin signaling in improving the root architecture and a theoretical basis for reasonable nitrogen fertilization during wheat production. Considering that the promotion of lateral root development by low nitrogen concentration has also been observed in maize [89], rice [90], cotton [91], rapeseed (*Brassica napus* L.) [95], and tea plant (*Camellia sinensis*) [131] and that larger root systems help the plant increase nitrogen uptake [33], we would strongly recommend a considerably lower rate of nitrogen fertilization at the seedling stage than conventional practices in crop productivity.

## Figures and Tables

**Table 1:** Root morphological parameters of wheat seedlings at 48 h after treatment.

Treatment	Root Tips	Total Root Length (cm)	Root Surface (cm^2^)
5 mM NO_3_^−^ (CK)	103.60 ± 17.32 b	123.85 ± 19.60 b	12.28 ± 1.75 b
0.1 mM NO_3_^−^ (LN)	150.02 ± 25.49 a	149.04 ± 15.61 a	14.01 ± 1.53 a
0.1 mM NO_3_^−^ + 60 mg/L TIBA (LNT)	61.84 ± 20.00 c	77.32 ± 11.23 c	8.48 ± 0.93 c

Values with different letters are significantly different at *p* < 0.05 among the treatments. Values represent the mean ± SD of nine replicates.

**Table 2:** List of genes regulating IAA biosynthesis and transport in the roots.

Gene ID	Gene Expression 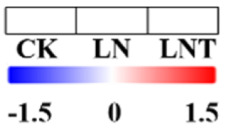			Gene Description	Function
TraesCS3A02G341400				Protein PIN-LIKES 6	Regulating the spatial redistribution of IAA in roots [39].
TraesCS2A02G430400				Auxin-responsive protein SAUR71	Regulating cell expansion downstream of auxin transport and auxin signaling [40].
TraesCS6B02G044300				High-affinity nitrate transporter 2.1	Polar auxin transport [35,36].
novel.9662				High-affinity nitrate transporter 2.1	
TraesCS6D02G035900				High-affinity nitrate transporter 2.1	
TraesCS2A02G323900				Putative multidrug resistance protein	Polar auxin transport [41].
TraesCS4A02G370600				Putative multidrug resistance protein	
TraesCS2D02G198800				Putative polyol transporter 2	Transcription factors of auxin biosynthesis and key molecular triggers for de novo organ patterning during LR formation [42,43].
TraesCS2A02G188900				Putative polyol transporter 2	
TraesCS2D02G199100				Putative polyol transporter 5	
TraesCS5A02G141700				Ethylene-responsive transcription factor WRI1	Regulating primary root growth through alterations in auxin homeostasis [44,45].
novel.7503				Ethylene-responsive transcription factor WRI1	
TraesCS5B02G002500				Agamous-like MADS-box protein AGL21	Positive regulation of auxin accumulation [46,47].
novel.12069				Agamous-like MADS-box protein AGL21	
TraesCS5D02G359700				Fanconi anemia group J protein homolog	Regulation of auxin output [48].
TraesCS5A02G352700				Fanconi anemia group J protein homolog	
TraesCS5B02G354900				Fanconi anemia group J protein homolog	
TraesCS7A02G373800				Transcription factor HY5	Negatively affecting *PIN3* and *LAX3* levels [49]. Transcription factors of brassinosteroid [50]; upregulating the expression of *TAA1* and *YUC5/7/8* [51].
TraesCS2A02G187800				BES1/BZR1 plant transcription factor	
TraesCS2D02G199900				BES1/BZR1 plant transcription factor	
TraesCS3A02G123500				BES1/BZR1 plant transcription factor	
TraesCS5A02G233700				Protein indeterminate-domain 14	Binding to promoter regions of *YUC5*, *TAA1*, and *PIN1* to activate their expression [52].
TraesCS5D02G240600				Protein indeterminate-domain 14	
TraesCS4A02G082200				Serine/threonine-protein kinase WAG1	Controlling the phosphorylation of *PIN* proteins [53].
TraesCS3A02G425700				Probable histone acetyltransferase HAC-like 1	Targeting auxin influx carrier *LAX2* and inducing auxin-responsive genes [54,55].
TraesCS5D02G193200				Histone acetyl transferase HAT1	
TraesCS5B02G186000				Histone acetyl transferase HAT1	

**Table 3:** DEGs coding for cell wall remodeling enzymes in the roots of wheat under different N treatments.

Gene ID	Gene Description	Log_2_ (FoldChange)	
		LN vs. CK	LNT vs. LN
TraesCS5D02G145900	Cellulase (glycosyl hydrolase family 5)	1.34	−3.08
TraesCS3A02G241000	Cellulase (glycosyl hydrolase family 5)	2.90	−2.23
TraesCS5D02G500000	Pectin acetylesterase 3	1.95	−6.42
TraesCS3D02G538500	Pectin acetylesterase 5	1.43	−6.25
TraesCS3A02G533800	Pectin acetylesterase 5	1.62	−7.31
TraesCS2B02G117500	Probable pectinesterase 53	2.76	−5.11
TraesCS1A02G143600	Probable pectate lyase 12	1.21	−1.28
TraesCS7B02G230600	Polygalacturonase	2.15	−3.45
TraesCS3B02G020300	Probable polygalacturonase	3.91	−7.45
TraesCS3D02G010500	Probable xyloglucan glycosyltransferase 3	1.44	−3.15
TraesCSU02G175000	Xyloglucan endotransglucosylase/hydrolase	2.40	−5.16
TraesCS2A02G498500	Xyloglucan endotransglucosylase/hydrolase 15	1.64	−5.56
TraesCS3B02G045700	Endo-1,4-beta-xylanase 1	2.55	−2.47
TraesCS4D02G227700	Endo-1,4-beta-xylanase 4	1.20	−2.45
TraesCS4B02G227000	Endo-1,4-beta-xylanase 4	2.55	−2.63
TraesCS3D02G039400	Xylanase inhibitor C-terminal	−2.13	3.56
TraesCS1D02G064500	Probable beta-1,4-xylosyltransferase	1.30	−1.05
TraesCS7B02G311200	Beta-1,2-xylosyltransferase	1.83	−5.36
TraesCS7D02G405200	Beta-1,2-xylosyltransferase	1.89	−3.93

**Table 4:** Expression of DEGs involved in the regulation of cell wall remodeling and LR formation.

Gene ID	Gene Expression 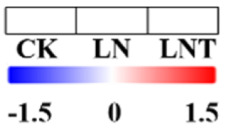			Gene Description	Function
TraesCS4B02G060000				Growth-regulating factor 6	Activating *OsYUC1* and auxin biosynthesis [56].
TraesCS4D02G059600				Growth-regulating factor 6	
TraesCS2A02G037100				Subtilisin-like protease SBT5.3	Activated by *NAC* and mediating auxin signaling to promote LR development by weakening connections among cells during root development [57].
TraesCS3B02G286900				Subtilisin-like protease SBT5.3	
TraesCS4B02G350600				NAC domain-containing protein 86	Transcription activator of genes involved in the biosynthesis of secondary cell walls, activating *AIR3*, and mediating auxin signaling to promote LR development [57].
andTraesCS3B02G439600				NAC domain-containing protein 48	
TraesCS7A02G068000				NAC domain-containing protein 43	
TraesCS4A02G421300				NAC domain-containing protein 43	
TraesCS2D02G199900				Brassinosteroid (BR)-regulated growth response	Inducing the expression of *cyclin D* genes in LR initiation and elongation [58].
TraesCS5A02G473800				APETALA2-like protein 3	Defining the primordium boundary during LR outgrowth [12].
TraesCS2D02G515800				APETALA2-like protein 5	
TraesCS3B02G251600				Tetratricopeptide repeat-containing thioredoxin TTL1	Regulating microtubule structure and cell wall elasticity [59].
TraesCS3B02G162100				Tetratricopeptide repeat-containing thioredoxin TTL1	
TraesCS6D02G305000				Glycosyl hydrolase family 17	Exhibiting glucan 1,3-beta-D-glucoside activity and relaxing the plant cell wall [41].
TraesCS3A02G374000				Glycosyl hydrolase family 17	
TraesCS4A02G005000				Plasma membrane ATPase 10	Excreting H^+^ ions into the cell wall compartment, lowering the cell wall pH, and initiating cell wall loosening and extended growth [60].
TraesCS4B02G300000				Plasma membrane ATPase 10	
TraesCS2D02G206300				Aquaporin PIP2-1	Promoting water transport from overlying tissue to the primordium [61,62,63].
TraesCS6A02G082900				Aquaporin PIP2-5	
TraesCS2B02G497800				CRIB domain-containing protein RIC10	Propagation of ROP GTPase signals [64].
TraesCS6D02G126000				CRIB domain-containing protein RIC10	
TraesCS4B02G113300				Protein NODULATION SIGNALING PATHWAY 2	Transcriptional regulator essential for Nod-factor-induced gene expression [65,66].
TraesCS4D02G110800				Protein NODULATION SIGNALING PATHWAY 2	

The three squares in the heatmap from left to right represent wheat seedlings treated with 5 mM NO_3_^−^ (CK), 0.1 mM NO_3_^−^ (LN), and 0.1 mM NO_3_^−^ + 60 mg/L TIBA (LNT), respectively.

**Table 5:** Effect of LN on the activity of root cell wall remodeling enzymes and the content of the main cell wall components.

	CK	LN	LNT
**Activity of cell-wall-remodeling-associated enzymes (μg min^−1^ g^−1^ FW)**			
pectinase	25.78 ± 0.30 b	34.97 ± 4.58 a	26.12 ± 1.37 b
β-1,3-glucanase	183.63 ± 7.95 b	735.34 ± 39.37 a	226.22 ± 11.30 b
Polygalacturonase	0.81 ± 0.03 c	3.32 ± 0.23 a	1.97 ± 0.16 b
exo-β-1,4-glucanase	19.85 ± 0.52 c	22.59 ± 0.92 a	21.16 ± 0.19 b
Cellulase	507.43 ± 3.94 b	576.20 ± 11.05 a	505.95 ± 12.57 b
neutral xylanase	96.84 ± 4.38 b	235.52 ± 23.28 a	117.03 ± 4.28 b
pectinate lyases	112.50 ± 4.69 b	152.71 ± 4.96 a	85.31 ± 3.57 c
Pectinesterase	43.19 ± 1.55 b	47.54 ± 2.08 a	44.65 ± 1.64 ab
**Cell wall components**			
galacturonic acid (μmol g^−1^ FW)	5.18 ± 0.16 b	5.70 ± 0.24 a	5.10 ± 0.23 b
cellulose (μmol g^−1^ FW)	4077.76 ± 45.73 a	2277.95 ± 76.69 c	2857.38 ± 40.26 b
DPM (%)	1.61 ± 0.06 a	1.32 ± 0.05 b	1.68 ± 0.06 a

The results represent the mean ± SD of at least three independent experiments. Different lowercase letters in each row indicate significant differences at *p* < 0.05. FW, fresh weight; DPM, degree of pectin-methyl esterification.

## Data Availability

The datasets used and/or analyzed during the current study are available from the corresponding author upon reasonable request.
